# Targeting NINJ1-mediated cell rupture to treat inflammatory diseases

**DOI:** 10.1186/s10020-025-01113-9

**Published:** 2025-02-14

**Authors:** Claire Ju-Eun Hur, Benjamin Ethan Steinberg

**Affiliations:** 1 Program in Neuroscience and Mental Health, The Hospital for Sick Children, Toronto, ON Canada; 2 Department of Physiology, University of Toronto, Toronto, ON Canada; 3 Department of Anesthesia and Pain Medicine, The Hospital for Sick Children, Toronto, ON Canada

**Keywords:** Cell death, Inflammation, Pyroptosis, Necrosis, Ferroptosis, NINJ1, Glycine, Muscimol

## Abstract

Cell death can terminate in plasma membrane rupture to release potent pro-inflammatory intracellular contents thereby contributing to inflammatory diseases. Cell rupture is an active process, mediated by the membrane protein ninjurin-1 (NINJ1) in pyroptosis, post-apoptosis lysis, ferroptosis, and forms of necrosis. Once activated, NINJ1 clusters into large oligomers within the membrane to initiate cellular lysis. Recent preclinical studies have demonstrated that inhibiting NINJ1 is a new strategy for treating immune-mediated diseases. Indeed, both small molecule inhibitors and neutralizing antibodies can target NINJ1 clustering to preserve plasma membrane integrity and mitigate disease pathogenesis. In this *Perspective*, we provide a summary of the current state of knowledge and recent developments in targeting cellular integrity during cell death through NINJ1 inhibition to treat inflammatory disease, with a focus on liver injury. As these NINJ1-mediated cell death pathways are pivotal in maintaining health and contribute to disease pathogenesis when dysregulated, the studies discussed within have broad implications across the immunologic basis of molecular medicine.

## Background

**Fig. 1:**
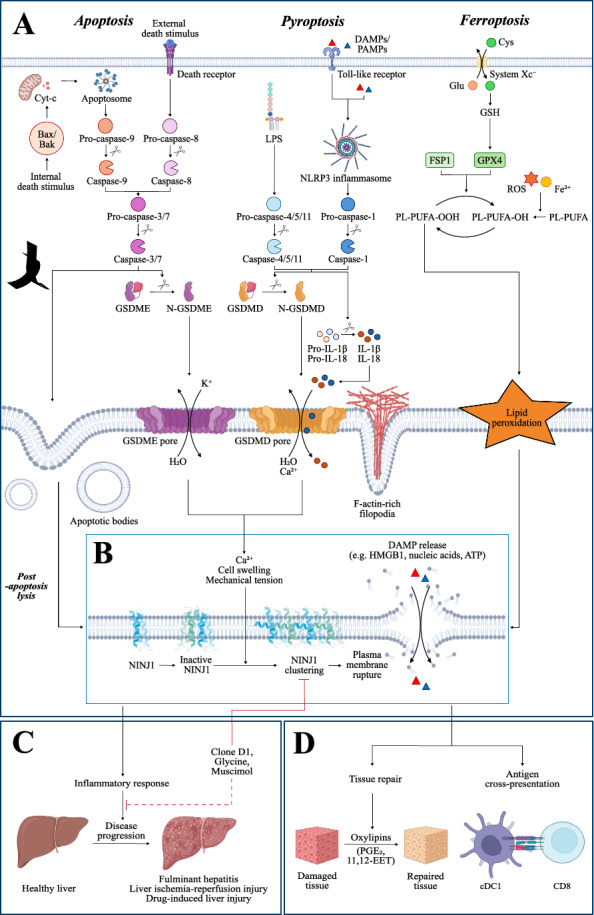
Mechanism and therapeutic targeting of NINJ1 in lytic cell death. **A** NINJ1 activation in apoptosis, pyroptosis, and ferroptosis. Apoptosis can be triggered by intrinsic and extrinsic pathways. In the intrinsic pathway, activated Bax and Bak oligomerize to mediate the permeabilization of the mitochondrial outer membrane, allowing for the release of cytochrome c (Cyt-c) into the cytoplasm. This leads to the activation of apoptosomes and the cleavage of pro-caspase-9 into the active initiator caspase-9. Caspase-9 cleaves and activates executioner caspase-3/7, which act on gasdermin E (GSDME). Cleaved GSDME oligomerizes within the plasma membrane to form a pore through which water enters, and potassium ions (K^+^) leave the cell. In the extrinsic pathway, an external death stimulus is recognized by a death receptor at the membrane that activates caspase-8 to in turn activate caspase-3/7. Apoptotic cells can form apoptotic bodies and be cleared by phagocytes through efferocytosis. Without efferocytosis, apoptotic cells undergo NINJ1-mediated plasma membrane rupture, termed post-apoptosis lysis. Pyroptosis is initiated by either canonical or non-canonical pathways. In the canonical pathway, damage-associated molecular patterns (DAMPs) or pathogen-associated molecular patterns (PAMPs) stimulate the assembly of inflammasomes, such as NLRP3, and activation of caspase-1. Caspase-1 processes interleukin-1 cytokines and cleaves gasdermin D (GSDMD). In the non-canonical pathway, intrinsic lipopolysaccharide (LPS) stimulates the cleavage of pro-caspase-4/5/11 to caspase-4/5/11, which cleaves GSDMD. Oligomerization of the N-terminus of GSDMD in the plasma membrane forms pores through which mature IL-1 cytokines leave and water and calcium enter the cell. Calcium entry can lead to filopodia formation that are decorated with filamentous actin (F-actin) and serve as a DAMP for type 1 conventional dendritic cells. Pyroptosis ultimately terminates in NINJ1-mediated plasma membrane rupture. In ferroptosis, the dedicated cysteine transporter system Xc^−^ imports cysteine (Cys) in exchange for glutamine (Glu). Cys is then converted to glutathione (GSH) which is used by glutathione peroxidase 4 (GPX4) to reduce phospholipid-polyunsaturated fatty acid-hydroperoxide (PL-PUFA-OOH) to PL alcohol (PL-PUFA-OH). Reactive oxygen species (ROS) and iron ions (Fe^2+^) contribute to the production of PL-PUFA-OH from phospholipid-polyunsaturated fatty acids (PL-PUFA), entering the reduction–oxidation cycle of PL-PUFA-OOH and PL-PUFA-OH. Ferroptosis suppressor protein 1 (FSP1) also contributes to the reduction of PL-PUFA-OOH. These reactions culminate in lipid peroxidation and NINJ1-mediated plasma membrane rupture. **B** All three lytic cell death mechanisms involve the activation of NINJ1. In resting cells, NINJ1 is found as an autoinhibited dimer. Upon activation, NINJ1 oligomerizes and the alpha helices within the N-terminus adopt a kinked configuration, leading to membrane rupture. Purported trigger of NINJ1 activation include calcium influx, cell swelling, and mechanical tension. Plasma membrane rupture allows for the release of DAMPs such as high mobility group box-1 (HMGB1), nucleic acids, and ATP into the extracellular space. **C** The detrimental effects of NINJ1-mediated lytic cell death. NINJ1-mediated plasma membrane rupture propagates inflammatory responses through the release of DAMPs. The inflammation contributes to disease pathogenesis, such as fulminant hepatitis, liver ischemia–reperfusion injury, and drug-induced liver injury. Anti-NINJ1 therapies that prevent NINJ1 clustering and cell rupture can limit disease progression. These therapies include a neutralizing antibody termed clone D1, the amino acid glycine, and the drug muscimol. **D** The beneficial effects of NINJ1-mediated lytic cell death. Pyroptosis can lead to the release of oxylipins, such as prostaglandin E_2_ (PGE_2_) and 11,12-epoxyeicosatrienoic acids (11,12-EET), that support tissue repair. NINJ1-mediated rupture in pyroptosis also contributes to antigen cross-presentation by type 1 conventional dendritic cells (cDC1) and enable the transition from innate to antigen-specific immune responses

Regulated cell death pathways are fundamental to the maintenance of a state of health (Newton et al. 2024). When dysregulated, they contribute to disease pathogenesis (Newton et al. 2024). For example, apoptosis maintains tissue homeostasis by removing damaged cells to limit the development of autoimmunity and malignancy whereas ineffective apoptotic programming may lead to tumor formation (Newton et al. 2024). Pyroptosis represents another genetically encoded pathway that is defined by cell death evoked by the gasdermin protein family (Galluzzi et al. 2018). The gasdermins are pore forming proteins through which interleukin-1 family cytokines are secreted from cells (Broz et al. 2020). In addition to cytokine secretion, pyroptosis is important for the clearance of intracellular pathogens (e.g. *Shigella* and *Salmonella*) and the propagation of inflammation in response to cellular damage or stress (Qu et al. 2016; Suzuki et al. 2014). Ferroptosis is a regulated form of cell death that involves iron-dependent lipid peroxidation (Dixon and Olzmann 2024), which can be activated in physiologic (e.g. tumour suppression) and pathophysiologic (e.g. neurodegeneration) conditions (Stockwell 2022). While these various cell death programs are initiated in response to different stimuli and serve biologically different functions in health and disease, they can each terminate in cellular rupture (Fig. 1A). This includes cells that have initiated apoptosis as they can undergo a post-apoptotic lysis, also termed secondary necrosis (Vanden Berghe et al. 2010). Cell rupture is a key pathological event as it results in the release of intracellular contents, namely danger associated molecular patterns (DAMPs) (Newton et al. 2024; Tang et al. 2023). Once released into the extracellular space, these DAMPs (e.g. high mobility group box-1, HMGB1) are recognized by cognate receptors to enhance the injurious inflammatory responses that propagate disease (Tang et al. 2023).

Until recently, the plasma membrane rupture associated with lytic cell death pathways was considered a passive event. It is now known that across multiple forms of lytic cell death (including pyroptosis, post-apoptosis lysis and ferroptosis; Fig. 1A), cellular rupture is an active process mediated by the transmembrane protein ninjurin-1 (NINJ1; Fig. 1B) (Kayagaki et al. 2021; Ramos et al. 2024; Dondelinger et al. 2023). Herein, the activation and structural basis for NINJ1-mediated membrane rupture, the use of NINJ1-targeted therapies in preclinical models of disease, and perspective on how limiting cellular rupture represents a *bona fide* strategy to treat inflammatory diseases are reviewed. We highlight both the injurious and beneficial physiologic effects of lytic cell death, and their ramifications on targeting NINJ1 as a therapeutic strategy to treat inflammatory disease. Throughout, we use liver injury models as the preclinical and clinical context for our discussion.

## Main text

First identified as a cell adhesion molecule (Araki and Milbrandt 1996), NINJ1 is a 16-kDa transmembrane protein largely conserved across Metazoans (Degen et al. 2023). It has a relatively broad expression profile, with a particularly high abundance in macrophages (Kayagaki et al. 2021). NINJ1 has two transmembrane helices (TM1 and TM2), and was postulated to have both its N and C termini residing extracellularly in the basal state (Kayagaki et al. 2021). The N-terminus contains two additional α-helices (AH1 and AH2) (Kayagaki et al. 2021; Pourmal et al. 2024). A recent cryogenic electron microscopy study of the inactive NINJ1 molecule presents the two proximal α-helices AH1 and AH2 as traversing the membrane to leave the N-terminus to reside in the cytoplasm (Pourmal et al. 2024). Of note, this structure of inactive NINJ1 precludes its ability to serve as a cell adhesion molecule as the N-terminus, which contains the purported adhesion domain, is not accessible from the extracellular side.

In resting macrophages, NINJ1 is thought to reside in low-order multimers within the plasma membrane (Fig. 1B) (Kayagaki et al. 2021; Borges, et al. 2022). Inactive NINJ1 exists as face-to-face homodimers, stabilized by polar contacts, with a 3-helix conformation and an unkinked transmembrane helix comprised of AH1 and AH2 (Pourmal et al. 2024). The membrane rupture-inducing hydrophilic surface remains hidden, suggesting *trans*-autoinhibition which is released upon stimulation by cell death signals (Pourmal et al. 2024).

Structural biology studies have also provided insight into the molecular mechanism by which NINJ1 generates membrane rupture (Degen et al. 2023; Sahoo et al. 2024; David et al. 2024). Upon activation of lytic cell death, NINJ1 clusters into large oligomers within the plasma membrane to induce cellular rupture (Fig. 1B) (Degen et al. 2023; Sahoo et al. 2024; David et al. 2024). Scanning mutagenesis studies identified the two N-terminal helices AH1 and AH2 as the functionally important domains for plasma membrane rupture (Kayagaki et al. 2021). While the underlying structural characteristics of the NINJ1 subunit are consistent across the structural studies, different models have been proposed as to how the NINJ1 cluster generates plasma membrane rupture (Degen et al. 2023; Sahoo et al. 2024; David et al. 2024). In their seminal work, Degen et al. show that NINJ1 polymerizes into structures that reach the micrometer scale and consist of branched and filamentous or ring-shaped assemblies (Degen et al. 2023). They propose that these morphologically diverse polymers generate cellular rupture through the N-terminal helices, which move into the plasma membrane to disrupt its integrity through formation of a lesion or pore within the membrane (Degen et al. 2023). Alternative models posit that NINJ1 oligomerizes into heterogeneous ring-like structures to destabilize the plasma membrane by either extracting membrane patches from the cell (David et al. 2024) or by warping around membrane protrusions to jettison them from the plasma membrane (Sahoo et al. 2024). Additional studies are necessary to reconcile these discrepant models of NINJ1-mediated plasma membrane rupture.

Moreover, the molecular trigger that activates NINJ1 to cluster and its modes of regulation remains unclear. A recent study proposes that cellular swelling is an activating stimulus (Dondelinger et al. 2023). This notion is consistent with the known volume expansion that occurs during pyroptosis and apoptosis; however, other cellular processes common to NINJ1-dependent lytic cell death pathways, such as ionic fluxes, may also be involved. For example, early data indicate that calcium is necessary for the aggregation of NINJ1 molecules and the subsequent rupturing of the membrane (Hartenian, et al. 2024; Borges, et al. 2024). Calcium influx may induce changes to the biophysical properties of the plasma membrane to induce NINJ1 clustering. This may be achieved by alterations in membrane tension and the induction of plasma membrane lipid scrambling by a calcium-activated phospholipid scramblase called TMEM16F (Borges, et al. 2024; Xu, et al. 2024). As noted above, NINJ1 remains quiescent in small autoinhibited homodimers in resting, viable cells (Pourmal et al. 2024). It stands to reason, that something is restraining NINJ1 from spontaneously clustering. What restricts NINJ1 is unknown and remains an important area of investigation as it may reveal a targetable regulatory process upstream of cell rupture.

One mechanism by which NINJ1-mediated plasma membrane rupture contributes to the progression of inflammatory diseases is the release of pro-inflammatory DAMPs (Fig. 1B, C). It follows that the inhibition of NINJ1 to limit plasma membrane rupture would therefore mitigate the injurious sequelae of pro-inflammatory DAMPs. While multiple DAMPs released by plasma membrane rupture have been described, we briefly highlight high mobility group box 1 (HMGB1), circulating cell free DNA (cfDNA), and filamentous actin as archetypes and contextualize their roles in inflammatory liver pathology. HMGB1 is a nuclear protein in healthy cells, which gets released into the extracellular environment through plasma membrane rupture (Volchuk et al. 2020). Extracellular HMGB1 signals through Toll-like receptors (TLR) and receptor for advanced glycation end products (RAGE) plasma membrane receptors to upregulate pro-inflammatory pathways (Andersson and Tracey 2011). For example, treatment with HMGB1 exacerbates liver ischemia–reperfusion-mediated hepatocellular injury, consistent with the correlation between HMGB1 levels during organ reperfusion and graft dysfunction in human liver transplant recipients (Terry et al. 2023). Circulating cfDNA is extracellular fragments of DNA released into bodily fluids through lytic cell death pathways such as apoptosis and necrosis (Rostami et al. 2020). The release material can be free or remain in complex with DNA-binding molecules like histones and HMGB1 (Marsman et al. 2016). These nucleic acids function as biomarkers of disease, including in graft dysfunction following liver transplantation (Levitsky et al. 2022). Notably, cfDNA may also serve as a pro-inflammatory DAMP through activation of nucleic acid sensing pathways such as cyclic GMP-AMP synthase (cGAS), which results in activation of stimulator of interferon genes (STING) to engage type 1 interferon immune activation (Hashimoto et al. 2021).

In contrast to the pro-inflammatory function of HMGB1 and cfDNA, other DAMPs may work to temper the injurious inflammation following lytic cell death (Fig. 1D). This is exemplified by the dendritic cell natural killer lectin group receptor-1 (DNGR-1; gene name *CLEC9A*), a plasma membrane receptor on dendritic cells that can recognize filamentous (F)-actin exposed from ruptured cells (Sancho et al. 2009; Ahrens et al. 2012; Zhang et al. 2012). Recognition of F-actin on dendritic cells mediates cross-presentation of dead cell-associated antigens to increase the efficiency of cytotoxic T cell priming (Fig. 1D). However, stimulation of DNGR-1 also decreases inflammatory cytokine and chemokine production to limit neutrophil recruitment to sites of sterile inflammation (Fresno et al. 2018), thereby mitigating tissue damage. F-actin-rich filopodia that are recognized by DNGR-1 have been described on the corpses of pyroptotic and necroptotic cells (Holley et al. 2025). While the formation of these F-actin-rich filopodia structures did not require plasma membrane rupture, NINJ1 was needed for them to engage the subsequent DNGR-1 signaling in dendritic cells (Holley et al. 2025). Together, these data indicate that NINJ1 allows for the activation of DNGR-1 signaling through F-actin exposure, which may both coordinate an innate to adaptive immune response transition and promote pro-tissue repair signals in certain contexts. These findings of potential pro-resolution effects of lytic cell death are complemented by recent studies that demonstrate that pro-resolution metabolites (e.g. oxylipins) can be released during pyroptosis to promote tissue repair (Fig. 1D) (Mehrotra et al. 2024; Chi et al. 2024). While these studies demonstrate that the oxylipin release is gasdermin-dependent, they do not rule out a contribution of NINJ1-driven terminal cell rupture.

Functionally, the identification of NINJ1 as the mediator of cell lysis is significant as NINJ1 has long been associated with the generation of inflammatory diseases through knockout and knockdown studies. These conditions include vascular diseases (Sheng et al. 2023; Wu et al. 2024), pancreatitis (Lee et al. 2023), and sepsis (Hartigh et al. 2023), each of which supports the involvement of NINJ1 in pathogenic inflammation. Herein, we focus on the multiple preclinical and clinical examples of NINJ1-driven liver injury. For example, in a mouse model of sterile fulminant hepatitis generated by tumor necrosis factor (TNF)-induced hepatocyte apoptosis, inducible genetic knockout of *Ninj1* limited liver injury, demonstrating a role for NINJ1 in apoptosis-related plasma membrane rupture in vivo and highlighting the functional importance of NINJ1 in hepatitis pathogenesis (Kayagaki et al. 2023). As compared with wildtype animals, *Ninj1* knockout mice had decreased serum markers of hepatocellular injury such as alanine aminotransaminase and aspartate aminotransferase, as well as lactate dehydrogenase, a general marker of membrane rupture (Kayagaki et al. 2023). These data are consistent with a mouse model of acute liver failure induced using lipopolysaccharide and D-galactosamine. In this model, whole animal *Ninj1* knockout animals had decreased circulating transaminase levels and inflammatory cytokine levels as compared with wildtype animals (Kim et al. 2022). These findings were attributed to the activation of NINJ1 within hepatocytes and not liver resident macrophages as myeloid-specific *Ninj1* knockout animals were not protected from injury (Kim et al. 2022).

In addition to toxin-induced inflammatory diseases, NINJ1 has been implicated in the sterile inflammation that results from ischemia–reperfusion injury (IRI) as seen during solid organ transplantation. IRI is a two-phased phenomenon in which initial tissue injury and death result from oxygen and nutrient deprivation due to ischemia, followed by sterile inflammation causing immune cell recruitment and activation during organ reperfusion (Martins et al. 2024). Liver IRI, for example, is a major cause of both acute and chronic organ dysfunction following liver transplant (Martins et al. 2024). NINJ1 deficiency within Kupffer cells (the resident macrophages of the liver) reduced neutrophil infiltration, intrahepatic inflammation, and hepatocyte apoptosis following liver IRI in a mouse model (Hu et al. 2023).

As of now, there is limited high-quality evidence for a role of NINJ1 from human studies; however, there is accumulating data associating NINJ1 with human diseases and inflammation. Germane to this *Perspective* on NINJ1-mediated cell rupture in liver injury, genome-wide association studies have identified a *NINJ1* single nucleotide polymorphism (rs7018885) that is associated with lower serum transaminase levels (Kayagaki et al. 2023; Bycroft et al. 2018; Sinnott-Armstrong et al. 2021). Conversely, increased *NINJ1* expression has been observed in liver samples from patients with acute liver failure and alcoholic hepatitis (Kim et al. 2022). As compared to control samples, elevated levels of NINJ1 have also been observed in other human conditions and diseases such as atrial fibrillation (Fang et al. 2022), lung tumorigenesis in the context of lung cancer (Hyun et al. 2022), and trophoblast cell dysfunction in the context of recurrent spontaneous abortion (Zhang et al. 2022). These data remain correlative and do not position NINJ1 directly in the pathogenesis of human disease. Given the growing body of evidence from preclinical and translation studies that implicate NINJ1 in disease pathogenesis and its inhibition as a therapeutic strategy, there is now increasing motivation to investigate NINJ1 in human disease.

In parallel to the above-described *Ninj1* knockout studies, preclinical investigations have also demonstrated that NINJ1-mediated cell rupture can be pharmacologically targeted, thereby paving the way for intervention trials in preclinical models of disease. The amino acid glycine has long been known to exert cytoprotective effects (Weinberg et al. 2016) but its mechanism of action was obscure. It is now appreciated that glycine prevents NINJ1 clustering within the plasma membrane to maintain cellular integrity across a variety of cell injury models (Borges, et al. 2022). Whether glycine acts directly on NINJ1 oligomers to limit their aggregation or via an intermediate remains to be explored. A computational approach that interrogated low-order NINJ1 oligomers suggest that glycine may stabilize NINJ1 dimers and therefore limit its ability to organize into the large clusters required for cell rupture (Sahoo et al. 2024). Given the potentially limiting pharmacokinetics of glycine, repositioning other approved medications for the purpose of targeting NINJ1 offers an alternative strategy. To that end, a recent study identified muscimol, a well-characterized GABA_A_ receptor agonist, as an inhibitor of NINJ1 mediated membrane rupture that was protective in a model of lipopolysaccharide-induced septic shock model (Hartigh et al. 2023). Both glycine and muscimol are unfortunately limited by a combination of poor pharmacokinetics and off-target effects. The identification of more specific and well-tolerated small molecule inhibitors of NINJ1 would rapidly advance the field and accelerate the translation of NINJ1 targeting into the clinical forum.

The above highlights that NINJ1 activation and clustering can be pharmacologically targeted and speak to the possibility of devising more specific and efficacious inhibitors. To circumvent the potentially limiting non-specificity of glycine or muscimol, NINJ1-specific targeting has been achieved in translational studies using an anti-NINJ1 neutralizing antibody (termed clone D1). Identified through a functional screen of 217 anti-mouse NINJ1 monoclonal antibodies, clone D1 was characterized as a NINJ1-specific inhibitory antibody which likely sterically interferes with NINJ1 oligomerization in the plasma membrane by engaging extracellular C-terminal residues (Kayagaki et al. 2023). In a series of translational studies, clone D1 was trialed as a therapeutic in a variety of acute liver injury models, including drug-induced fulminant hepatitis and liver IRI (Kayagaki et al. 2023). Anti-NINJ1 therapy suppressed injury and systemic inflammatory responses as evidenced by decreased serum markers of hepatocyte injury and circulating markers of pro-inflammatory cell rupture (e.g. HMGB1), respectively (Kayagaki et al. 2023).

These studies evaluated anti-NINJ1 therapy in acute and sterile inflammatory injury models. Whether a comparable strategy will work against a chronic injury disease process should be explored. This will require a detailed assessment of potential negative consequences of long-term NINJ1 inhibition given the recently defined beneficial effects of lytic cell death pathways in tissue repair processes and the transition from innate to adaptive immunity (Holley et al. 2025; Mehrotra et al. 2024; Chi et al. 2024). Understanding both the positive and negative ramifications of inhibiting cell rupture to maintain a state of health (e.g. tissue repair) or combatting disease (e.g. tumor immunity) represents a key area of future research for the field. Lastly, a specific antibody or small drug effective against human NINJ1 will need to be developed as clone D1 shows no significant reactivity against the human protein. These will be important next steps in developing therapies that target NINJ1-induced plasma membrane rupture in disease pathogenesis.

## Conclusion

In summary, targeting NINJ1-mediated cell rupture to treat disease is now a viable therapeutic strategy for exploration across a variety of inflammatory diseases. For example, the protective effect of NINJ1 inhibition on liver IRI should encourage future work directed at targeting NINJ1 for tissue preservation during solid organ transplantation in which lytic cell death is a major contributor to poor outcomes. A better understanding of the mechanisms of activation and regulation of NINJ1-mediated cell rupture may identify additional molecules and cellular processes that can be targeted therapeutically to treat disease.

## Data Availability

No datasets were generated or analysed during the current study.

## Abbreviations

cfDNA: Cell free DNA
cGAS: Cyclic GMP-AMP synthase
CLEC9A: C-Type lectin domain containing 9A
DAMP: Danger associated molecular pattern
DNGR-1: Dendritic cell natural killer lectin group receptor-1
GABA: Gamma-aminobutyric acid
GSDMD: Gasdermin D
HMGB1: High mobility group box 1
IRI: Ischemia–reperfusion injury
NINJ1: Ninjurin-1
RAGE: Receptor for advanced glycation end products
STING: Stimulator of interferon genes
TLR: Toll-like receptors
TNF: Tumor necrosis factor
